# Neurological outcomes and associated perinatal factors in infants born between 22 and 25 weeks with active care

**DOI:** 10.1038/s41372-024-02093-0

**Published:** 2024-09-18

**Authors:** Yu Ariyoshi, Takayuki Iriyama, Takahiro Seyama, Seisuke Sayama, Eriko Yano, Kensuke Suzuki, Taiki Samejima, Mari Ichinose, Masatake Toshimitsu, Kenbun Sone, Atsushi Ito, Yoshihiko Shitara, Keiichi Kumasawa, Kohei Kashima, Satsuki Kakiuchi, Yasushi Hirota, Naoto Takahashi, Yutaka Osuga

**Affiliations:** 1 Department of Obstetrics and Gynecology, Faculty of Medicine, The University of Tokyo, 7-3-1 Hongo, Bunkyo-ku, Tokyo 113-8655 Japan; 2 Department of Pediatrics, Faculty of Medicine, The University of Tokyo, 7-3-1 Hongo, Bunkyo-ku, Tokyo 113-8655 Japan

**Keywords:** Paediatrics, Pregnancy outcome

## Abstract

**Objectives:**

To elucidate the outcomes of periviable infants receiving active care (AC) and explore perinatal factors associated with neurodevelopmental outcomes.

**Methods:**

This is a single-center retrospective study on infants born at 22–25 weeks of gestation, all of whom received AC. A developmental quotient (DQ) ≥ 85 at corrected 18 months was judged as normal.

**Results:**

Fifty-seven infants were included in the study. The survival rates at discharge were 83%, 86%, 93%, and 93% at 22, 23, 24, and 25 gestational weeks, respectively. The overall percentage of normal DQ was 26/47 (55%). Acidemia in the arterial blood gas measured within 6 h after birth was identified as a factor significantly associated with subnormal DQ.

**Conclusions:**

Not only high survival rates, but also favorable neurodevelopmental outcomes may be achieved by AC in periviable infants. Moreover, impaired neurodevelopmental outcomes may be associated with early postnatal acidemia following initial resuscitation.

## Introduction

An executive summary of proceedings from a joint workshop held in 2013 defines “periviable birth” as delivery occurring between 20 0/7 through 25 6/7 weeks of gestation [1]. Families and healthcare teams face many challenges when delivery is anticipated during the periviable period [2]. Advances in perinatal medicine have significantly improved the survival rates of periviable infants in recent years [3, 4]. However, the decision to provide active care (AC)— generally including obstetric and neonatal management such as antenatal steroids, cesarean birth, neonatal resuscitation, and respiratory support—to infants born at 22–23 weeks of gestation remains controversial. While AC can enhance the survival rates of extremely premature infants compared to palliative care [5], increased survival rates may be associated with a higher risk of neurodevelopmental impairment among survivors [6]. Guidelines in some countries recommend individualized care for infants at 22-23 weeks of gestation, but AC is not widely recommended [7–10]. Consequently, studies investigating the effect of AC on survival rates and neurodevelopmental outcomes are limited.

Furthermore, in studies examining the survival rates of newborns receiving AC starting at 22 weeks of pregnancy, the percentage of cases subjected to such resuscitation shows significant variability. In a 3-year population-based cohort study conducted in Sweden [11], among infants born at 22, 23, 24, and 25 weeks of gestation, AC was performed at rates of 38%, 81%, 93%, and 98%, respectively, and the one-year survival rates for those that received AC were 26%, 65%, 73%, and 84%, respectively. In a comprehensive cohort study in the United States [12], AC was provided to 29,932 cases, and the survival rates at 22, 23, 24, and 25 weeks gestational ages were 39%, 55%, 71%, and 83%, respectively. Notably, the analysis excluded 3540 cases that received palliative care. There is limited reporting regarding AC provided to the entire research cohort of periviable infants.

Furthermore, identifying clinical factors that impact subsequent neurological outcomes in surviving periviable infants is crucial. Postnatal complications related to prematurity, including bronchopulmonary dysplasia (BPD), have been associated with impaired neurological outcomes in extremely preterm infants [13, 14]. However, the relationship between perinatal clinical factors up to early postnatal stages and neurodevelopmental outcomes in periviable infants remains unclear.

Since Japan modified the threshold for initiating AC from 24 weeks to 22 weeks in 1991, there has been a discernible trend toward more frequent AC from 22 weeks of gestational age [15]. A recent survey of almost all perinatal centers across Japan reported notably high rates of AC: 85% at 22 weeks of gestation and 98% at 23 weeks of gestation [16]. As a policy of our institution as a tertiary care center, we have made clinical decisions based on a thorough, shared decision-making (SDM) process with the parents when deliveries were expected around the periviable period, especially at 22 weeks of gestation. Subsequently, parents desired AC in all cases, including antenatal steroid administration, cesarean section with fetal indications, and respiratory support after delivery; thus, we have managed each case accordingly. Here, we aimed to elucidate the survival and neurodevelopmental outcomes of all periviable infants who received AC and examine the perinatal clinical factors that may influence their neurodevelopmental outcomes.

## Methods

This study was approved by the Institutional Review Board at the University of Tokyo (Approval Number: 2022023NI). Clinical data were collected from the medical records of 66 mother-child pairs who were managed and delivered at 22–25 weeks of gestation at the University of Tokyo Hospital between January 2011 and December 2020. Infants with intrauterine fetal death, twin pregnancies, congenital infections, chromosomal abnormalities, or infants with congenital anomalies were excluded. This retrospective observational study was conducted using the opt-out method on our hospital website, as per the guidance of the ethics committee and guidelines.

### Active care in our hospital

When delivery was anticipated after 22 0/7 weeks of gestation, we confirmed the parents’ desire for AC after thoroughly sharing the decision-making process. Antenatal steroids were administered at 12 mg of betamethasone every 24 h, ideally administered twice, starting after 22 0/7 weeks of gestation if the delivery was expected within one week. Cesarean sections were performed based on fetal concerns such as abnormal presentation, non-reassuring fetal status, and suspected intraamniotic infection. However, cesarean sections at 22 weeks of gestation were performed after SDM process with the parents based on a thorough assessment of each case. Two or more obstetricians that were board-certified specialists performed all cesarean sections, and a neonatologist was present during all procedures. As standard practice, all infants underwent resuscitation in the delivery room or operation room and were subsequently admitted to the neonatal intensive care unit (NICU) after birth. Every infant was intubated immediately after delivery, and surfactant was administered soon after intubation or after arriving at the NICU, depending on its necessity. Treatment approaches and management for periviable births have remained consistent over the past decade at our institution.

### Neurodevelopmental assessment

Experienced neonatologists and trained clinical psychologists assessed the neurodevelopment at 18 months of corrected age. The Developmental Quotient (DQ), calculated by dividing the developmental age by the corrected age and multiplying by 100, was determined using the Kyoto Scale of Psychological Development (KSPD) [17]. The KSPD was used as the neurodevelopmental assessment tool, evaluating three domains: postural-motor, cognitive-adaptive, and language-social domains. Each domain was assessed individually, and an overall Developmental Quotient (DQ) was calculated. The mean DQ was 100.6 with a standard deviation of 13.4 [18]. When the KSPD was unavailable, the Kinder Infant Development Scale (KIDS) and Enjoji Developmental Scale, both of which are standardized for use with Japanese infants, were employed. The KIDS and Enjoji Developmental Scale are developmental screening questionnaires that assess domains such as motor, language, and social relationships, and provide an overall DQ. As with KSPD, DQ ≥ 85 is considered normal in these tests. Therefore, we classified a total DQ ≥ 85 as normal and DQ < 85 as subnormal in this study, consistent with previous literature [19–22]. These tests were conducted by well-trained pediatricians or psychologists. Cerebral palsy, defined as a non-progressive disorder of movement, posture, and motor function [23], was classified as subnormal DQ. Cases classified as cerebral palsy included all levels of Gross Motor Function Classification System (GMFCS).

### Maternal and fetal characteristics

The gestational age was accurately determined using the date of the last menstrual period with confirmation through an obstetric ultrasound examination in the first trimester. The delivered placenta underwent pathological examination, and the presence of an in utero infection was defined as either stage II or higher histological chorioamnionitis or funisitis, based on Blanc’s criteria [24]. Preeclampsia was diagnosed according to the International Society for the Study of Hypertension in Pregnancy requirements [25]. Using Japanese anthropometric charts, infants whose birth weight was below the 10th percentile for their gestational age were defined as small for gestational age [26]. Bronchopulmonary dysplasia (BPD) was defined as the requirement of oxygen and/or mechanical ventilator support or continuous positive airway pressure at 36 weeks of postmenstrual age [27]. Intraventricular hemorrhage (IVH) and periventricular leukomalacia (PVL) were determined based on early postnatal brain ultrasound findings and magnetic resonance imaging conducted in all cases at discharge [28, 29]. IVH was classified as grade 3 or higher, and PVL included both cystic and non-cystic PVL. Necrotizing enterocolitis (NEC) was defined as stage 2 according to the Bell criteria [30]. Retinopathy of prematurity (ROP) was defined as cases requiring treatments such as anti-VEGF therapy or photocoagulation [31].

### Blood gas analysis

Umbilical arterial blood gas (ABG) samples were collected from the clamped umbilical cord immediately after birth by experienced obstetricians, and the umbilical ABG pH was promptly measured. Whenever possible, the ABG samples from the umbilical catheter or peripheral arterial line were assessed after admission to the NICU. The first ABG sample collected in the NICU was referred to as “first ABG.” In addition, the number of ABG samples collected within the first 24 h of birth and their gas parameters were recorded. The lowest values of ABG pH, along with the associated base excess (BE), pO2, pCO2, and HCO3- values, were recorded at 6 h intervals to assess temporal changes in ABG parameters. Acidemia in the umbilical ABG was defined as pH < 7.0 [32], and acidemia in the ABG was defined as pH < 7.2 [33]. Infants with metabolic acidosis who were treated with sodium bicarbonate within 24 h after birth were also screened. The decision for and timing of sodium bicarbonate administration were determined by the pediatrician.

### Statistical analysis

The Mann–Whitney U test was used for continuous variables and Fisher’s exact test for nominal variables. Adjusted odds ratios (ORs) and 95% confidence intervals (CIs) were calculated using logistic regression models to assess the effect of clinical factors on neurodevelopment, with gestational age considered a confounding factor. All tests were two-tailed, and a significance level of *p* < 0.05 was used to determine statistical significance. Statistical analyses were performed using EZR, which provides a graphical user interface for R (R Foundation for Statistical Computing, Vienna, Austria) [34].

## Results

**Fig. 1:**
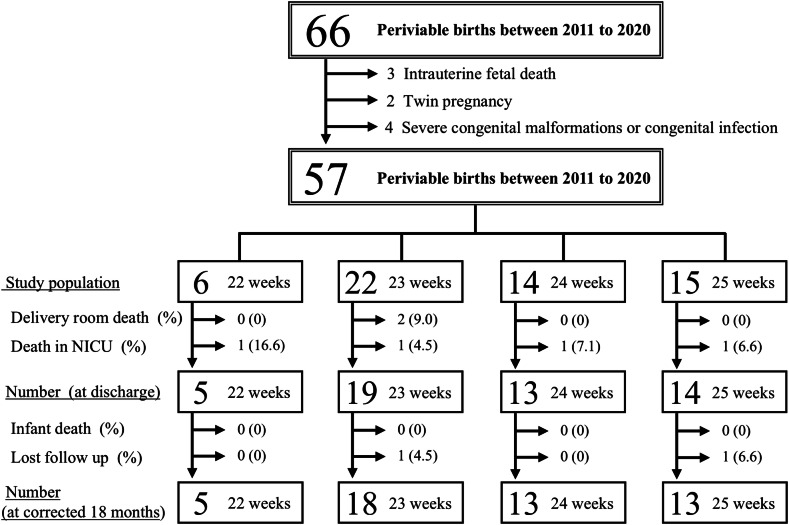
Flow diagram depicting infant details. NICU, neonatal intensive care unit.

A flowchart of the study design is shown in Fig. 1. A total of 57 infants met the inclusion criteria for this study, all of whom received AC after birth; none received palliative care. The survival rate at discharge was 51/57 (89%): two infants died in the delivery room immediately after birth and four in the NICU prior to discharge. No deaths occurred after NICU discharge. The follow-up rate at 18 months was 49/51 (96%). Neurodevelopmental assessment was conducted in 49 patients at a corrected median of 18 months [17, 18, 35]. The assessment methods included KSPD for 31 cases, KIDS for 8 cases, and the Enjoji developmental scale for 6 cases. Among them, 4 (9%) were diagnosed with cerebral palsy and all of them had DQ < 85. Two infants with KSPD underwent neurodevelopmental assessment; however, the DQ could not be measured because the children were uncooperative.

Table 1 shows the types of AC provided to newborns and the neurodevelopmental outcomes categorized by gestational age. Tracheal intubation was performed in all patients. Surfactant treatment was administered to all infants, except for one at 23 weeks and one at 25 weeks of gestation. The cesarean section rate was 50% at 22 weeks of gestation and 86% overall. The rates of normal DQ at corrected 18 months were 50% (2/4) at 22 weeks, 50% (9/18) at 23 weeks, 42% (5/12) at 24 weeks, and 77% (10/13) at 25 weeks.

**Table 1:** The types of active care and neurodevelopmental outcomes categorized by gestational age.

	Infants, number (%)									
	22 wk	*n*	23 wk	*n*	24 wk	*n*	25 wk	*n*	Total	*n*
Active care										
One or two doses of antenatal steroids	6 (100)	6	17 (77)	22	11 (79)	14	11 (73)	15	45 (79)	57
Cesarean delivery	3 (50)	6	19 (86)	22	14 (100)	14	13 (87)	15	49 (86)	57
Tracheal intubation	6 (100)	6	22 (100)	22	14 (100)	14	15 (100)	15	57 (100)	57
Surfactant treatment	6 (100)	6	21 (95)	22	14 (100)	14	14 (93)	15	55 (96)	57
Survival rate										
at discharge	5 (83)	6	19 (86)	22	13 (93)	14	14 (93)	15	52 (91)	57
at corrected 18 months	5 (83)	6	18 (86)	21	13 (93)	14	13 (93)	14	50 (90)	55
Neurodevelopmental assessment at corrected 18 months										
Normal DQ	2 (50)	4	9 (50)	18	5 (42)	12	10 (77)	13	26 (55)	47

*DQ* developmental quotient.

Normal DQ was defined as DQ ≥ 85.

To investigate the perinatal clinical factors that may influence neurodevelopment, we compared normal and subnormal DQ (Table 2). There were no differences between the normal and subnormal DQ that were associated with maternal background or the incidence of maternal complications. Placental analysis revealed no significant differences in the prevalence of in utero infections between the two groups. There were no differences in gestational age, birth weight, or other background factors that could be associated with differences in DQ scores. In the subnormal DQ, the proportion of 5 min Apgar scores <7 was significantly higher when compared to that in the normal DQ (81% vs. 42%; *p* < 0.001). The umbilical ABG pH did not differ between the two groups. However, the pH of first ABG was significantly lower in the subnormal DQ (7.215 [7.108–7.283] vs. 7.340 [7.233-7.386]; *p* = 0.01). There were 2 cases (4%) where ABG was not obtained: one case had a normal DQ, and the other case had a subnormal DQ. The time from delivery to the first ABG sampling did not differ between the groups (normal DQ: 118 [78–218] vs. subnormal DQ: 92 [78–103]; *p* = 0.15). In addition, in the subnormal DQ, cases of metabolic acidosis treated with sodium bicarbonate within 24 h were significantly higher than that in the normal DQ (86% vs. 54%]; *p* = 0.02). The relationships between postnatal factors and subsequent neurological outcomes are shown in the supplementary data. Regarding neonatal complications, the incidence of BPD was significantly higher in the subnormal DQ group (85% vs. 50%; *p* = 0.02). The incidences of other typical neonatal complications, such as intraventricular hemorrhage (IVH), did not differ between the two groups.

**Table 2:** Comparison of perinatal clinical factors between normal and subnormal DQ.

	Infants, number (%)				
	Normal DQ	*n*	Subnormal DQ	*n*	*P*
Maternal characteristics					
Maternal age, median (IQR), y	36 (32–39)	26	32 (29–36)	21	0.09
Body mass index>25 kg/m^2^	3 (12)	26	4 (19)	21	0.68
Assisted reproductive technology	4 (15)	26	4 (19)	21	>0.99
Multiparous	13 (50)	26	6 (29)	21	0.23
Smoking history	1 (4)	26	4 (19)	21	0.15
One or two doses of antenatal steroid	21 (81)	26	15 (71)	21	0.50
Preterm premature rupture of membranes	11 (42)	26	7 (33)	21	0.56
In utero infection	13 (50)	26	13 (62)	21	0.56
Preeclampsia	1 (4)	26	2 (10)	21	0.57
Placental abruption	1 (4)	26	2 (10)	21	0.57
Infant characteristics					
Male sex	11 (42)	26	10 (48)	21	0.77
Gestational age, median (IQR), wk	24.3 (23.6–25.1)	26	23.6 (23.1–24.4)	21	0.07
Birth weight, median (IQR), g	605 (534–728)	26	565 (504–658)	21	0.12
Small for gestational age	6 (23)	26	5 (26)	21	0.73
Postnatal assessment					
1 min Apgar score <4	11 (42)	26	10 (48)	21	0.56
5 min Apgar score <7	11 (42)	26	17 (81)	21	<0.001
Umbilical ABG pH, median (IQR)	7.292 (7.203–7.367)	20	7.329 (7.185–7.369)	20	0.96
Umbilical ABG pH < 7.0	1 (5)	20	2 (10)	20	>0.99
First ABG pH, median (IQR)	7.340 (7.233–7.386)	25	7.215 (7.108–7.283)	20	0.01
First ABG pH < 7.2	4 (16)	25	10 (50)	20	0.02
Sampling time of First ABG, median (IQR), min	118 (78–218)	25	92 (78–103)	20	0.15
Metabolic acidosis treated with sodium bicarbonate within 24 h	14 (54)	26	18 (86)	21	0.02

*ABG* arterial blood gas, *DQ* developmental quotient, *IQR* interquartile range.

For the perinatal factors that exhibited significant differences in univariate analysis, we conducted logistic regression analyses with the outcome variable as subnormal DQ and adjusted for gestational age at birth, respectively. Logistic regression analysis revealed that 5 min Apgar scores <7 (ORs [95%CI]; 4.7 [1.2–19.5]), first ABG pH < 7.2 (ORs [95%CI]; 4.4 [1.1–18.4]), and metabolic acidosis treated with sodium bicarbonate within 24 h (ORs [95%CI]; 4.4 [1.0–19.4]) were associated with significantly increased odds of having subnormal DQ, regardless of gestational age (Table 3).

**Table 3:** Logistic regression analyses with subnormal DQ as the outcome variable.

	Unadjusted			Adjusted ^a^		
Perinatal factors	OR	95%CI	*P*	OR	95%CI	*P*
5-minute Apgar score <7	5.8	1.5–22.1	0.01	4.7	1.2–19.5	0.03
First ABG pH < 7.2	5.3	1.3–20.9	0.01	4.4	1.1–18.4	0.03
Metabolic acidosis treated with sodium bicarbonate within 24 h	5.1	1.2–21.8	0.02	4.4	1.0–19.4	0.04

^a^Adjustments were made for gestational age.

*DQ* developmental quotient, *ABG* arterial blood gas, *OR* odds ratio, *CI* confidence interval.

**Fig. 2:**
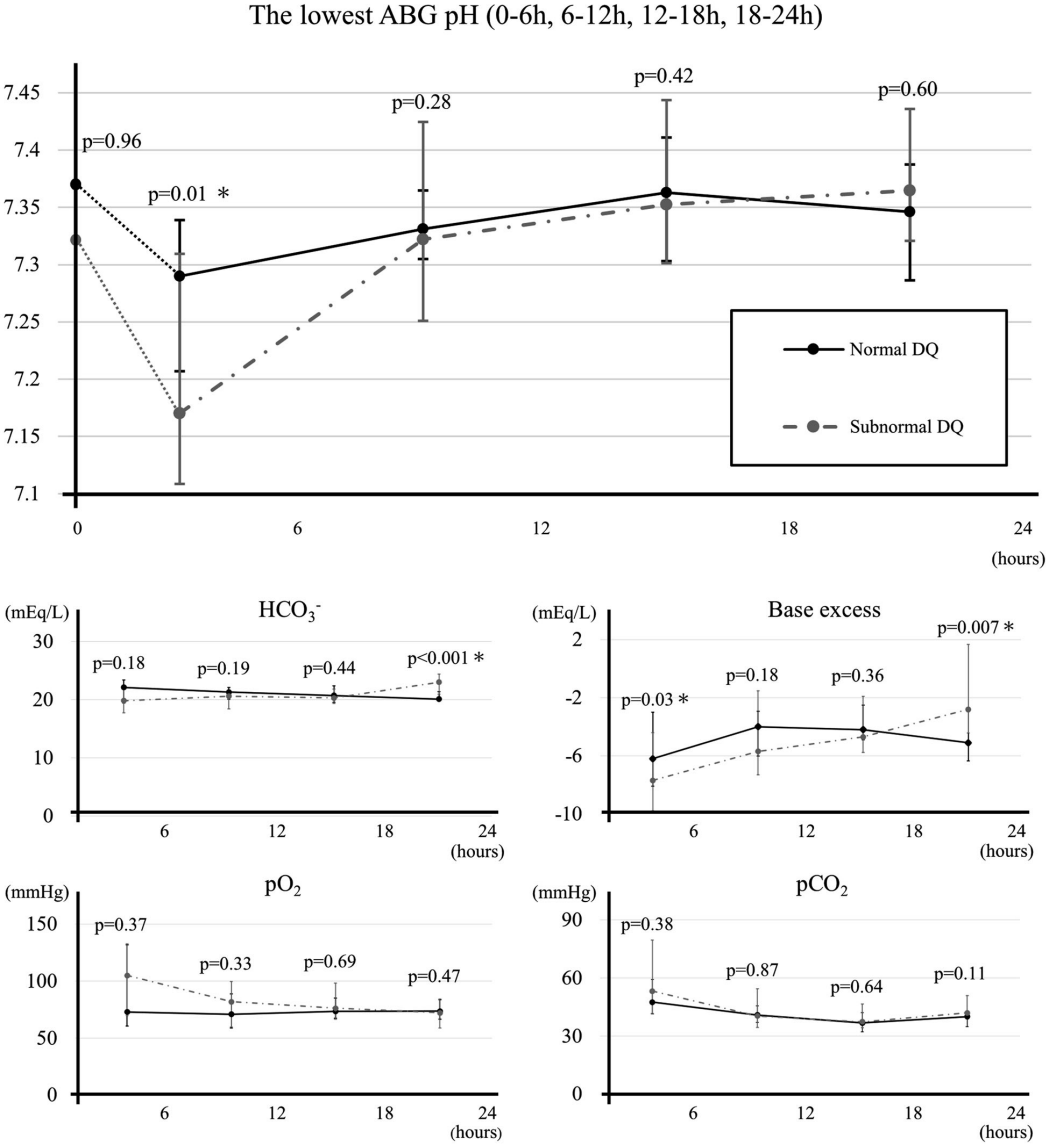
A chart depicting the minimum arterial blood gas pH and the corresponding gas parameters recorded at 6-hour intervals after birth, categorized as 0–6 h (*n* = 20, 19), 6–12 h (*n* = 22, 20), 12–18 h (23, 20), and 18–24 h (*n* = 23, 20). (*n*=normal DQ, subnormal DQ). At 0 h, the umbilical artery blood gas pH is included as a reference value. Data are presented as median [interquartile range]. Statistical significance was set at p < 0.05 (*). DQ developmental quotient.

We analyzed ABG parameters within 24 h of birth in the normal and subnormal DQ to further investigate the relationship between ABG and neurodevelopment (Fig. 2). In the subnormal DQ, the minimum pH value of the ABG during 0–6 h after birth was significantly lower than that in the normal DQ (7.170 vs. 7.291; *p* = 0.01). However, the difference in postnatal ABG parameters between the two groups disappeared over time. During 0–6 h after birth, the BE values were significantly lower in the subnormal group, suggesting more severe metabolic acidosis ( − 7.7 vs. −6.2; *p* = 0.03). Conversely, during the 18–24 h period, the normal DQ had significantly lower BE values (−2.8 vs. −5.1; *p* = 0.007) and lower HCO3- levels (23.0 vs. 20.1; *p* < 0.001). However, the two groups had no significant differences in ABG pH (7.364 vs. 7.346; *p* = 0.60). In addition, the subnormal DQ had a higher frequency of ABG evaluations, with a median of three assessments within 6 h (compared with two in the normal DQ, *p* = 0.04) and a median of eight assessments within 24 h (compared to seven in the normal DQ, *p* = 0.01).

## Discussion

AC for periviable infants resulted in high survival rates and positive neurodevelopmental outcomes. Furthermore, our study is the first to suggest a potential link between early postnatal acidemia in periviable infants and later neurodevelopmental impairments.

AC for periviable births is known to improve infant survival rates [5]; however, it remains controversial owing to potential maternal complications [36, 37] and long-term outcomes for the infants [6, 38]. Recently, there has been increasing attention on SDM for periviable births [39, 40]. It is essential to respect the preferences and choices of patients and their families when considering AC. Over the past 10 years, we have conducted prenatal and postnatal counseling at our facility, explaining all potential risks; consequently, all parents opted for AC. To date, there have been few studies reporting survival rates and subsequent neurodevelopmental outcomes for cohorts where all periviable births received AC. Thus, we believe our study contributes new evidence to these discussions.

The high survival rate observed in the present study may be attributed to high rates of antenatal steroid administration and cesarean sections. The use of antenatal steroids significantly improves survival rates and reduces complications in periviable births, particularly in infants aged 22–23 weeks [12]. Additionally, compared to vaginal delivery, cesarean section has been reported to reduce the mortality rates and risk of IVH in extremely premature infants [41, 42]. Our study had a high rate of antenatal steroid administration (79%) and cesarean section deliveries (86%), which may have contributed to favorable outcomes. Similarly, Haga et al. [43] reported a high survival rate of 84% when AC was provided to all cases starting at 22 weeks of gestation, with a high cesarean section rate of 87%.

While concerns about neurodevelopmental outcomes for survivors persist when AC is applied in all cases, our study reported favorable results. To date, there are few studies that have undertaken AC across all cases. Studies by Kyser et al. [44] and Watkins et al. [45] have reported rates of 66% and 73% normal neurological development at 18 months, respectively, but these studies excluded infants receiving palliative care. Another study in Japan that performed AC in all cases reported a rate of 53% normal neurological outcomes at age 6; however, this study included gestational weeks from 22 to 27 and had a low follow-up rate [43]. Our study, with its high follow-up rate, likely reflects the most realistic outcomes seen thus far. While our results contribute a positive perspective to the discussion on AC and subsequent neurological outcomes, further research is necessary.

Most previous studies on the survival rates of actively resuscitated neonates excluded infants receiving palliative care, potentially leading to selection bias and not accurately representing actual population survival rates [12, 46, 47]. Our entire study cohort with AC demonstrates that even in periviable infants, survival rates can be favorable with comprehensive care strategies. These include prenatal steroid administration, cesarean delivery for fetal indications, and postnatal resuscitation. Given the improved survival rates in periviable births, the next goal of perinatal medicine should be to ensure that survivors lead their subsequent lives without impaired neurodevelopmental outcomes. To achieve this, we focused on perinatal clinical factors that affect neurological outcomes from the obstetric perspective.

Our study further established a correlation between acidemia in the first ABG sample obtained in the NICU and the occurrence of impaired neurodevelopmental outcomes at corrected 18 months. The first ABG reflects the responsiveness to initial resuscitation and is of distinct significance compared to the umbilical ABG, which measures the fetal metabolic status and oxygenation at the time of delivery immediately after birth. Furthermore, we focused on the ABG parameters within 24 h of birth and demonstrated a significant association between the low blood gas pH measured within 6 h after birth and the corresponding low BE and DQ values at corrected 18 months. Leviton et al. [33, 48] published two studies on the relationship between postnatal ABG pH and subsequent development. They reported that ABG disturbances over the first three days after birth may induce brain injury and affect neurological outcomes. Our study supports these findings, and our results focus more on the time course of blood gas parameters. In our study, infants with subnormal DQ at 18 months corrected age showed a higher usage rate of sodium bicarbonate and underwent frequent blood gas analyses within 24 h of birth. Interestingly, infants with subnormal DQ had higher BE and HCO3- levels 18–24 h after birth. The potential impact of uncontrollable, sustained metabolic acidosis from birth on subsequent neurodevelopmental outcomes requires attention. If periviable infants respond well to postnatal resuscitation and are successfully stabilized, it suggests potentially favorable neurological outcomes. Achieving this requires appropriate management during pregnancy to ensure better conditions at birth and comprehensive perinatal care which seamlessly transitions from obstetrics to pediatrics, ensuring readiness for neonatal resuscitation. Although the detailed mechanisms of acidemia and neurological outcomes are not well understood, our findings provide a new perspective to improving neurodevelopmental outcomes.

The strengths of our study are as follows. By performing AC in all cases, this study allows for a more accurate evaluation of the impact of AC on the outcomes of periviable infants compared to past reports. Additionally, due to the high follow-up rate after birth, the impact of dropouts was minimized. This study also has several limitations. First, this was a retrospective, single-center study with relatively few cases. We did not adjust for some factors such as sex, birth weight, or use of antenatal steroids in the multivariate analysis. Furthermore, in our study, known factors associated with subnormal DQ, such as GA, BW, IVH, and PVL [49, 50], were not shown to have significant associations. Additionally, our study identified the lowest DQ at 24 weeks, with these findings possibly attributable to the small number of cases. Moreover, our results may not be generalizable because the threshold of viability varies significantly by country and region. Second, standardized neurodevelopmental assessment methods are required. Many previous studies have utilized the Bayley Scale, whereas at our institution, different methods were employed based on the study period. Additionally, while multiple examiners conducted neurological tests at our facility, we did not evaluate inter-rater reliability. Furthermore, sociodemographic factors, such as maternal years of education, are known to impact neurological outcomes of preterm infants [51]; however, our study did not investigate these factors. Third, this study focused on neurodevelopment up to the corrected age of 18 months. It is generally known that DQ at 18–24 months does not necessarily correlate with later DQ [13]. Thus, future research should investigate subsequent neurodevelopment and include long-term follow-up of neurodevelopmental outcomes.

In conclusion, we achieved a high survival rate with AC for periviable infants. Additionally, we demonstrated for the first time a potential association between early postnatal acidemia in periviable infants and subsequent impaired neurodevelopmental outcomes. These findings could help improve future outcomes for periviable infants.

## Supplementary information

Supplementary data

## Data Availability

The datasets analyzed during the current study are available from the corresponding author on reasonable request.
