# Supplementary material for: Neurological outcomes and associated perinatal factors in infants born between 22 and 25 weeks with active care
Source: J Perinatol. 2024 Sep 18;45(2):186–93. doi: 10.1038/s41372-024-02093-0 (PMC11825359; doi:10.1038/s41372-024-02093-0)
Supplement: Supplementary file 1 — Supplementary data [file 41372_2024_2093_MOESM1_ESM.docx]

**Supplementary data.** Comparison of postnatal factors between normal and subnormal DQ.

|  | Infants, number (%) | | | | |
| --- | --- | --- | --- | --- | --- |
|  | Normal DQ | n | Subnormal DQ | n | P |
| Postnatal factors |  |  |  |  |  |
| Bronchopulmonary dysplasia | 12 (50) | 24 | 17 (85) | 20 | .02 |
| Intraventricular hemorrhage | 2 (8) | 26 | 3 (14) | 21 | .64 |
| Periventricular leukomalacia | 1 (4) | 26 | 5 (24) | 21 | .07 |
| Necrotizing enterocolitis | 2 (8) | 26 | 2 (10) | 21 | >.99 |
| Retinopathy of prematurity | 15 (63) | 24 | 14 (67) | 21 | >.99 |

DQ, developmental quotient
